# Understanding the blood‐brain barrier breakdown and vascular dysfunctions in new AD mouse models

**DOI:** 10.1002/alz.093393

**Published:** 2025-01-03

**Authors:** Yuanpu Chiu

**Affiliations:** ^1^ USC, Los Angeles, CA USA

## Abstract

**Background:**

Alzheimer’s disease (AD), the leading cause of dementia accounting for 70% of cases, involves complex pathogenesis with amyloid, tau, and cerebrovascular dysfunction contributing significantly. Vascular changes and impairment are strongly associated with AD pathogenesis in new knock‐in models from the MODEL‐AD consortium – early‐onset AD (EOAD) and late‐onset AD (LOAD) mice – which can be influenced by spatial transcriptomic genetic factors.

**Method:**

We will thoroughly characterize vascular dysfunction in these models over time: 3, 6, 9 months for EOAD mice; 4, 8, 12 months for LOAD mice. Age‐matched C57BL/6J mice will serve as controls. Additionally, we will apply cutting‐edge spatial transcriptomics with subcellular resolution (Stereo‐seq) to one EOAD and one LOAD model to uncover molecular changes underlying neurovascular dysfunction. We will integrate the obtained data with existing resources.

**Result:**

The pathological phenotypes of the new AD mouse models are expected to express microglial responses that are positively correlated with amyloid beta aggregation, leading to tau pathology. Vasculature is also expected to be impaired. The distribution of the phenotypes tends to be in cortical and hippocampal regions.

**Conclusion:**

Our outcomes will provide an enhanced understanding of blood‐brain barrier (BBB) breakdown and vascular contributions in physiological AD models, guiding future mechanistic and therapeutic studies of vascular‐directed strategies to mitigate neurodegeneration.

